# Somatic Versus Cognitive Depressive Symptoms as Predictors of Coronary Artery Disease among Women with Suspected Ischemia: The Women’s Ischemia Syndrome Evaluation

**DOI:** 10.4103/hm.hm_34_21

**Published:** 2021-11-30

**Authors:** Ashley S. Emami, C. Noel Bairey Merz, Jo-Ann Eastwood, Carl J. Pepine, Eileen M. Handberg, Vera Bittner, Puja K. Mehta, David S. Krantz, Viola Vaccarino, Wafia Eteiba, Carol E. Cornell, Thomas Rutledge

**Affiliations:** 1 Psychology Service, VA San Diego Healthcare System, San Diego, California,; 2 Barbra Streisand Women’s Heart Center, Cedars-Sinai Smidt Heart Institute, Los Angeles, California,; 3 UCLA School of Nursing, Los Angeles, California,; 4 Department of Medicine, Division of Cardiovascular Medicine, University of Florida, Gainesville, Florida,; 5 Department of Medicine, Division of Cardiovascular Disease, University of Alabama at Birmingham, Birmingham, Alabama,; 6 Department of Medicine, Division of Cardiology, Emory University School of Medicine, Atlanta, Georgia,; 7 Department of Medical and Clinical Psychology Uniformed Services University, Bethesda, Maryland,; 8 Department of Epidemiology, University of Pittsburgh, Pittsburgh, Pennsylvania,; 9 Department of Health Behavior and Health Education, University of Arkansas for Medical Sciences, Little Rock, Arkansas,; 10 Department of Psychiatry, University of California, San Diego, California

**Keywords:** Beck Depression Inventory, coronary artery disease, depression, ischemia, women

## Abstract

**Background:**

Depression is an established predictor of coronary artery disease (CAD) progression and mortality. “Somatic” symptoms of depression such as fatigue and sleep impairment overlap with symptoms of CAD and independently predict CAD events. Differentiating between “somatic” and “cognitive” depressive symptoms in at-risk patients may improve our understanding of the relationship between depression and CAD.

**Methods:**

The study utilized data from the Women’s Ischemia Syndrome Evaluation. Participants (*N* = 641; mean age = 58.0 [11.4] years) were enrolled to evaluate chest pain or suspected myocardial ischemia. They completed a battery of symptom and psychological questionnaires (including the Beck Depression Inventory [BDI]) at baseline, along with quantitative coronary angiography and other CAD diagnostic procedures. The BDI provided scores for total depression and for cognitive and somatic depressive symptom subscales.

**Results:**

Two hundred and fourteen (33.4%) women met criteria for obstructive CAD. Logistic regression models were used to examine relationships between depression symptoms and obstructive CAD. Neither BDI total scores (odds ratio [OR] =1.02, 95% confidence interval [CI], 0.99–1.05, *P* = 0.053) nor BDI cognitive scores (OR = 1.02, 95% CI, 1.00–1.04, *P* = 0.15) predicted CAD status. BDI somatic symptom scores, however, significantly predicted CAD status and remained statistically significant after controlling for age, race, and education (OR = 1.06, 95% CI, 1.01–1.12, *P* = 0.02).

**Conclusion:**

Among women with suspected myocardial ischemia, somatic but not cognitive depressive symptoms predicted an increased risk of obstructive CAD determined by coronary angiography. Consistent with prior reports, these results suggest a focus on somatic rather than cognitive depressive symptoms could offer additional diagnostic information.

## Introduction

Coronary artery disease (CAD) remains the leading cause of death for adults in the United States.^[1]^ Traditional risk factors for CAD include hypertension, dyslipidemia, diabetes, obesity, and nicotine dependence.^[1,2]^ Beyond these traditional risk factors, there is evidence to suggest that psychological factors such as depression should also be considered in the prevention, evaluation, and treatment of CAD.^[3]^

Depression is an established predictor of CAD progression and premature mortality.^[4]^ Patients with CAD and co-occurring depression are also more likely to have inadequately managed CAD risk factors and poor treatment adherence; each may lead to adverse outcomes.^[5]^ Depression rates are higher among women than men^[6]^ – including among women with established CAD – suggesting that women with CAD and depression could be more vulnerable to CAD events.^[4]^ While risk factor management and treatment adherence should be monitored in all CAD patients, close monitoring and follow-up are especially important for patients with depression.^[7]^ Finally, a large body of epidemiological literature has linked depression to CAD events, suggesting that the assessment of depression may improve the diagnosis of CAD in clinical care settings. Particularly for women presenting with cardiac symptoms – among whom traditional symptoms and some diagnostic testing methods (e.g., treadmill exercise tests) for obstructive CAD are less accurate compared to men^[8]^ – assessing the presence and pattern of depressive symptoms could augment existing diagnostic algorithms.

When examining the link between depression and CAD, there is growing empirical evidence that subtypes of depressive symptoms should be considered. “Somatic” symptoms of depression (e.g., fatigue and sleep impairment) overlap substantially with symptoms of CAD and independently predicted CAD events relative to “cognitive” symptoms (e.g., loss of interest and pessimism) in several cohort studies.^[9–11]^ Although previous research supports relationships between somatic symptoms of depression and the risk of subsequent CAD events,^[12]^ whether these somatic symptoms are also associated with CAD status or CAD severity is unknown. Therefore, our paper aimed to contribute to the growing research in this area by investigating relationships between total depressive symptoms, cognitive depressive symptoms, and somatic depressive symptoms with objectively diagnosed CAD presence and severity in a sample of women with suspected myocardial ischemia. Based on previous research, we hypothesized that somatic but not cognitive depressive symptoms would be associated with the presence of angiographically defined obstructive CAD in a sample of women referred for coronary angiography to evaluate chest pain or suspected myocardial ischemia. Second, we aimed to compare the predictive value of the Beck Depression Inventory (BDI) somatic symptom subscale with CAD status to established risk factors for CAD to examine the clinical significance of the relationship.

## Methods

### Participants

The purpose of the Women’s Ischemia Syndrome Evaluation (WISE) was to improve the understanding and diagnosis of ischemic heart disease in women. An extensive description of the WISE protocol and methodology has been previously published.^[8]^ Women (≥18 years old) undergoing a clinically indicated coronary angiogram to evaluate signs and symptoms of CAD were recruited for the original cohort from four centers (University of Alabama at Birmingham, University of Florida, Gainesville, University of Pittsburgh, and Allegheny General Hospital, Pittsburgh) beginning in 1996, using inclusion and exclusion criteria published previously.^[8]^ All participants provided written informed consent, and all participating sites obtained Institutional Review Board approval.

### Measurement of coronary artery disease and coronary artery disease risk factors

The WISE Angiographic Core Laboratory (Rhode Island Hospital, Providence, RI) performed qualitative and quantitative analysis of coronary angiograms, with investigators blinded to all other subject data.^[13]^ Coronary atherosclerosis was quantified using a modified Gensini angiographic severity score (hereafter referred to as an angiographic severity score).^[14]^ This severity score was developed with points assigned according to the category of severity of the stenosis (0–19, 20–49, 50–69, 70–89, 90–98, 99–100) adjusting for partial and complete collaterals. Scores were further adjusted according to lesion location, with more proximal lesions receiving a higher weighting factor. From the angiogram results, we defined coronary disease severity for each participant as “nonobstructive CAD” (<50% angiographic severity score) or “obstructive CAD” (≥50% angiographic severity score). Among 641 WISE participants with complete angiography, CAD risk factor, and depression data, 33.4% were determined to have obstructive CAD.

Major cardiovascular disease risk factors included smoking status (never/former or current), hypertension, dyslipidemia, diabetes, and body mass index (BMI <30 or ≥30 kg/m^2^). We defined hypertension, dyslipidemia, and diabetes status for this report on the basis of participant report of a history of treatment for these conditions (lifestyle or medication).

### Demographic variables

Demographic variables included participant age, education (≤ high school education or >high school education), and race (Caucasian or non-Caucasian). Demographic variables were used as control variables in the logistic regression analyses.

### Depression symptoms

Participants completed the BDI, a 21-item measure of depressive symptoms. The BDI is a widely used and validated depression measure previously described in the WISE sample.^[15]^ Table 1 summarizes the somatic and cognitive depressive symptom content from the BDI.

### Statistical analyses

Frequencies and descriptive statistics were used to analyze the demographic, BDI, and cardiac risk factor data. BDI subscale scores (cognitive and somatic) and total scores were computed. We also compared women above and below the median score of five on the BDI somatic subscale. Independent samples *t*-tests were used to assess differences between women with obstructive and nonobstructive CAD on BDI scores, as well as differences between women with and without cardiac risk factors and cardiac risk factor medications (angiotensin-converting enzyme inhibitors, angiotensin receptor blockers, beta blockers, statins) on somatic depression symptoms. Chi-square analyses were used to examine the associations between cardiac risk factors and somatic symptoms and CAD status. Pearson’s product-moment correlation coefficients were used to examine bivariate correlations between BDI scores (total and subscale scores) and angiographic severity scores.

Separate logistic regression models were conducted to examine relationships between depression symptoms (total and cognitive/somatic subscales) and obstructive CAD. Each of these separate logistic regression models were adjusted *a priori* for demographic variables including age, ethnicity, and education history. We calculated area under the receiver operator characteristic (ROC) curve values for each somatic and CAD risk factor predictor to assess model discrimination. ROC values ≤ 0.50 represent no greater than chance prediction. We also conducted an exploratory analysis to examine whether Caucasian and non-Caucasian participants differed on somatic depression symptoms. All statistical tests were completed using SPSS Statistics software version 26.0 (IBM, Chicago, IL, United States).

## Results

Table 2 provides a description of the WISE sample on pertinent demographics, cardiovascular risk factors, and BDI scores. The sample was middle-aged, primarily Caucasian, and more than 40% showed evidence of clinically significant depression based on BDI scores ≥10.

**Figure 1:**
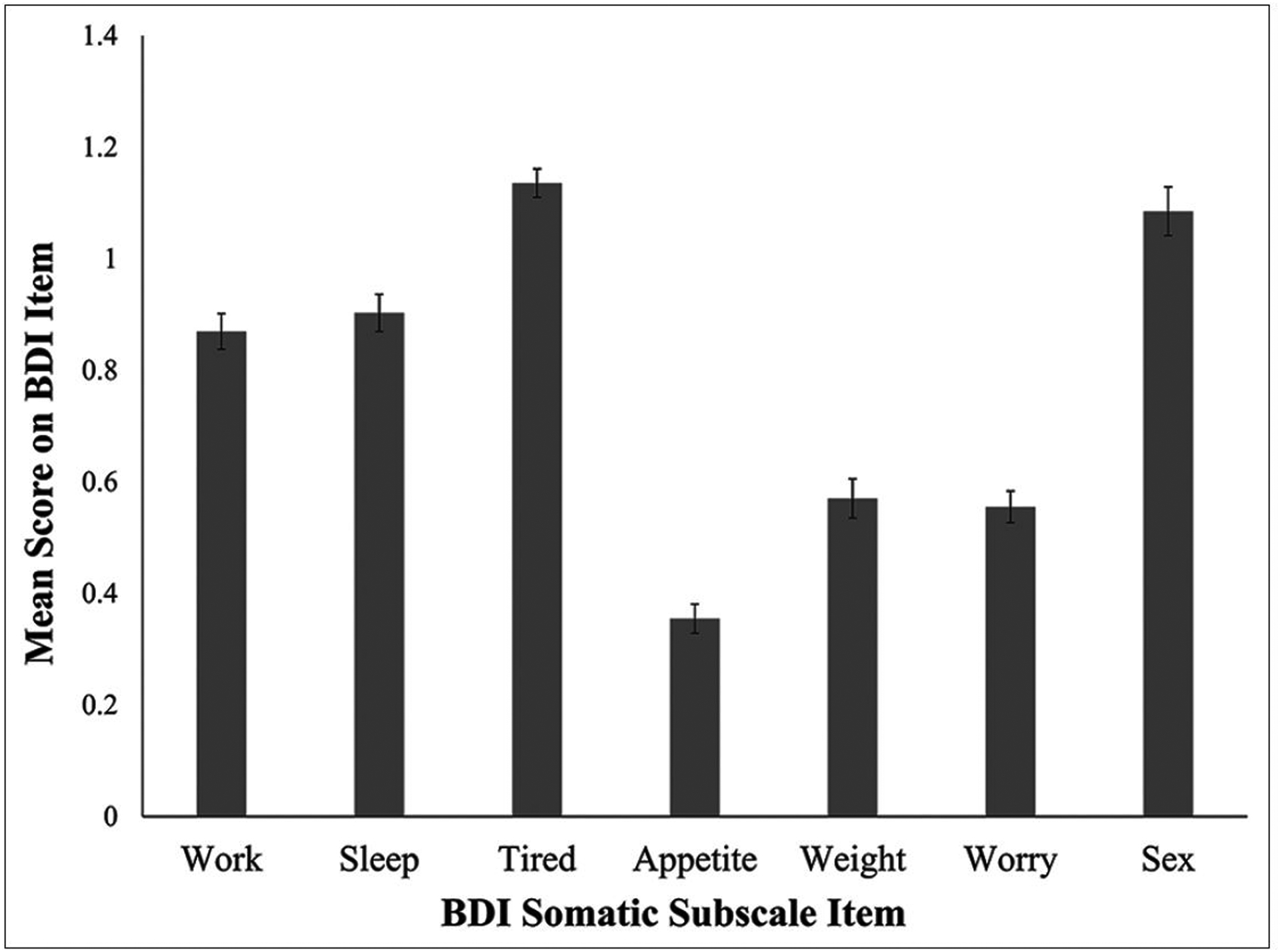
Item level means for Beck Depression Inventory somatic subscale items. Note: Error bars indicate the standard error of the mean

Figure 1 displays item level means for the seven BDI somatic subscale items. Items 15, 16, 17, and 21 (difficulties related to work, sleep, fatigue, and sex, respectively) had the highest item level means for the total sample. This pattern for item level means was similar for women with and without obstructive disease.

### Beck Depression Inventory scores and coronary artery disease status

BDI somatic symptoms scores were significantly associated with obstructive CAD (odds ratio [OR] =1.06, 95% confidence interval [CI], 1.01–1.12), and the relationship remained statistically significant after controlling for age, race, and education (*P* = 0.02). Each point increase on the BDI somatic subscale was associated with a 6% increased risk of having obstructive CAD. Total BDI scores (OR = 1.02, 95% CI, 0.99–1.05, *P* = 0.053) and BDI cognitive scores (OR = 1.02, 95% CI, 1.00–1.04, *P* = 0.15) were nonsignificant CAD predictors. Similarly, across the full sample (i.e. not stratified by CAD status), BDI somatic subscale scores predicted angiographic severity scores, *r* (629) =0.09, *P* = 0.03 but BDI total (*r*[629] =0.03, *P* = 0.48) and cognitive subscale (*r*[629] = −0.01, *P* = 0.79) scores were not significantly correlated with angiographic severity scores.

Participants with obstructive and nonobstructive CAD were also compared on BDI total and subscale scores [Table 2]. There was a significant difference between the groups on BDI somatic subscale scores, *t* (387) =2.48, *P* = 0.01. In contrast, the groups did not differ on BDI cognitive subscale scores (*t*[639] =0.28, *P* = 0.78) or BDI total scores (*t*[639] =1.23, *P* = 0.17).

An exploratory analysis also demonstrated that somatic subscale scores were significantly higher for non-Caucasian participants (*M* = 6.72, standard deviation [*SD*] =3.67) than for Caucasian participants (*M* = 5.23 *SD* = 3.26), *t* (138) =3.89, *P* < 0.001.

### Somatic depression symptoms and cardiac risk factors as predictors of coronary artery disease status

**Figure 2:**
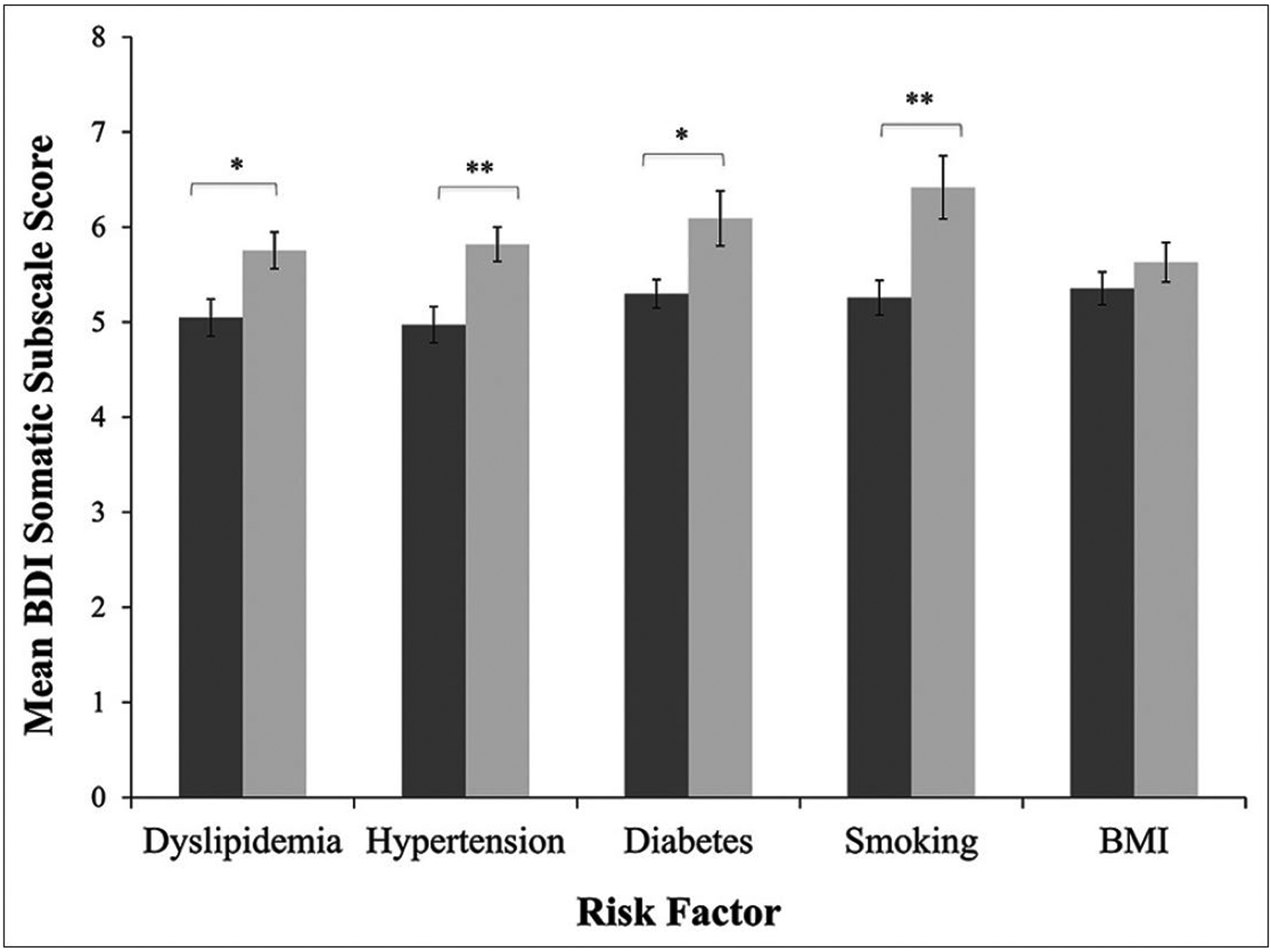
Risk Factor by Somatic Subscale Score. Note: *P<0.01, **P<0.05. Error bars indicate the standard error of the mean. These results are unadjusted for demographic variables. Women who did not have the risk factor are displayed in dark grey, while women with the risk factor are displayed in light grey

**Figure 3:**
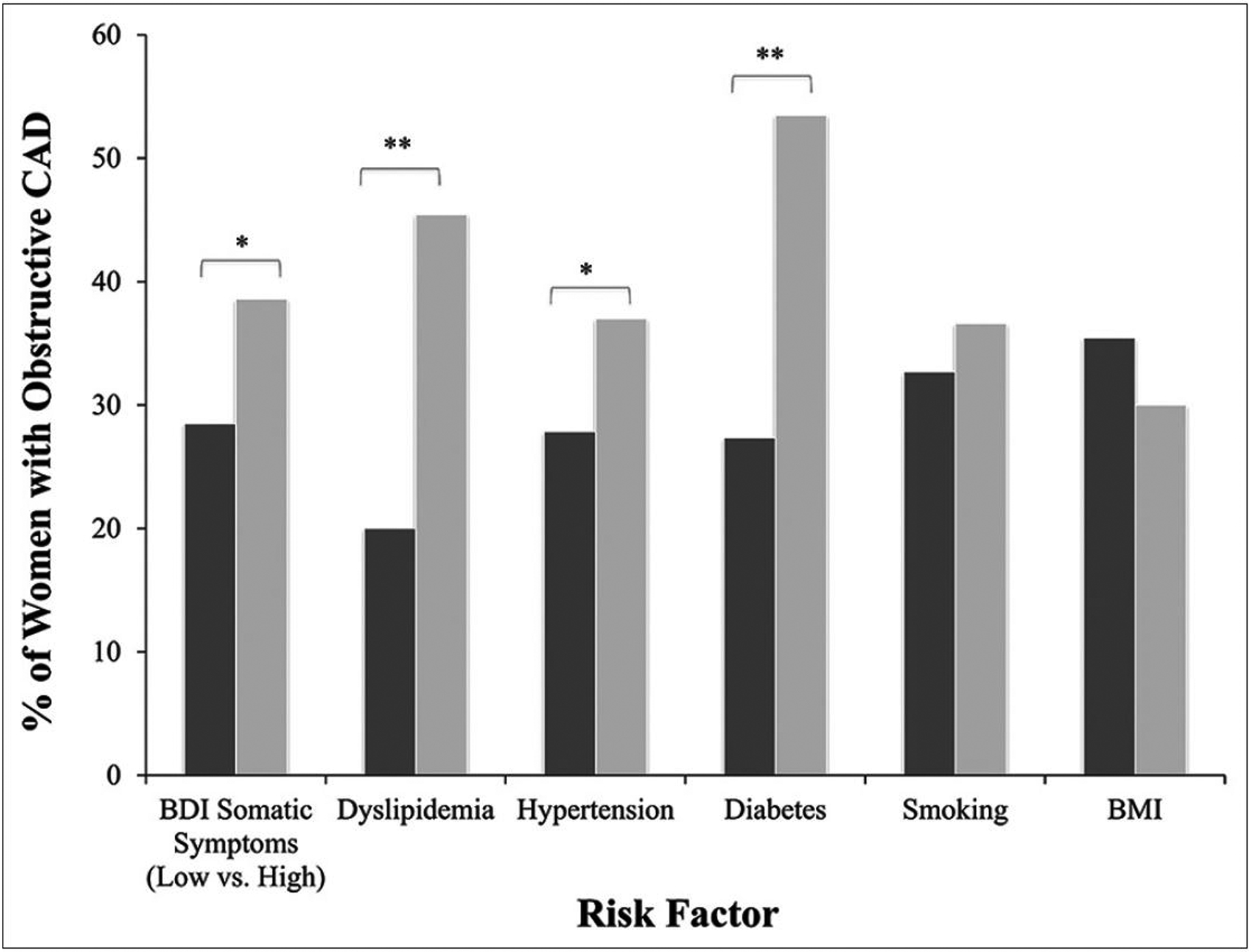
Rates of Obstructive CAD by Risk Factor. Note: **P<0.001, *P<0.05. These results are unadjusted for demographic variables. Women who did not have the risk factor are displayed in dark grey, while women with the risk factor are displayed in light grey.

Figure 2 displays mean somatic subscale scores for women with and without cardiac risk factors. Rates of obstructive CAD by risk factor are displayed in Figure 3. Table 3 provides results for the covariate-adjusted logistic regression analyses. In Step 1, demographic variables (age, race, and education) were entered into the model. In Step 2, each predictor was entered into the model to determine its unique contribution to predicting obstructive CAD status. In the separate models, somatic subscale scores, dyslipidemia, diabetes, and smoking status were significant predictors of obstructive CAD (*P* < 0.05). In contrast, hypertension and BMI did not significantly predict obstructive CAD in the demographic-adjusted regression models (*P* > 0.05). We also used the median-split somatic subscale variable to compare women. Women in the “high” group were at a significantly higher risk for obstructive CAD than women in the “low” group (*P* = 0.04) after covariate adjustment.

Finally, we assessed somatic symptom differences between women with and without cardiac risk factors. Women with dyslipidemia (*t*[600] =2.58, *P* = 0.01), hypertension (*t*[614] =3.22, *P* = 0.001), and diabetes (*t*[636] =2.49, *P* = 0.01) and current smokers (*t*[170] =3.21, *P* = 0.002) had significantly higher scores on the somatic depression symptoms subscale than women without these cardiac risk factors. Scores on the somatic depression symptoms subscale did not differ when the sample was compared based on BMI (<30 or ≥ 30), *t* (636) =1.00, *P* = 0.32.

We also performed independent *t*-tests for each of the four medication categories to assess differences in somatic symptoms. There were no significant differences for angiotensin-converting enzyme inhibitors (*t*[619]) =1.91, *P* = 0.06), angiotensin receptor blockers (*t*[619]) =0.91, *P* = .35), beta blockers (*t*[619]) =0.53, *P* = 0.60), and statins (*t*[620]) =1.02, *P* = 0.31).

## Discussion

Among a sample of women with suspected myocardial ischemia, somatic but not cognitive depressive symptoms were associated with obstructive CAD defined by coronary angiography. This finding is consistent with previous research that has identified somatic depressive symptoms as a more reliable predictor of cardiovascular events than cognitive depression symptoms.^[9–11]^ An earlier study with the WISE cohort^[9]^ found, for example, that somatic but not cognitive depressive symptoms were associated with an increased risk of cardiovascular-related mortality and events over 5.8 years of follow-up. Similarly, de Jonge *et al*.^[11]^ found that somatic depressive symptoms were associated with cardiovascular death and cardiac-related readmissions during an average follow-up of 2.5 years. Somatic depression symptoms remained predictive of poor cardiovascular prognosis even after baseline health factors were controlled. In contrast, cognitive depression symptoms were not associated with cardiovascular death and cardiac events in the latter study.

Our study builds on previous prospective studies suggesting value for a greater focus on somatic symptoms among patients with elevated CAD risk. Despite this supportive evidence, many standard measures of depression specifically exclude or minimize somatic content out of concern that it may conflate with physical illness. Measures such as the Hospital Anxiety and Depression Scale, Geriatric Depression Scale, and The Patient Health Questionnaire-9 are among those limiting somatic symptom content. The BDI (and BDI-II) include the most somatic content among validated depression questionnaires, and this may explain why these measures have been consistent in predicting CVD in many previous observational studies.

The focus on somatic depressive symptoms reinforces previous American Heart Association (AHA)^[3]^ recommendations for the assessment of depression in CAD patients while suggesting benefit from a more refined measurement approach. Screening measures for depression become useful when they improve patient outcomes above existing standards of care. Some research has questioned whether depression screenings in primary care and cardiac settings achieve this standard.^[16,17]^ Our findings align with and may offer a partial explanation for previously observed limitations of measuring global depressive symptoms in CAD populations. Most screening measures for depression used in hospital settings, for instance, have minimal somatic symptom content; this absence may weaken their utility to providers. Alternatively, the BDI or BDI-II could be administered to patients before appointments. The information yielded by the BDI somatic symptoms subscale may then assist providers in their risk assessment and diagnostic decision-making processes.

Previous research has identified several possible explanations as to why somatic depression symptoms may be a stronger predictor of cardiovascular outcomes relative to cognitive symptoms. One explanation is that somatic symptoms may be more strongly linked to HPA axis and sympathetic nervous system-mediated biological mechanisms thought to underlie the depression-CAD relationship. For example, Stewart *et al*.^[18]^ found that somatic symptoms of depression, but not the cognitive symptoms, predicted 6-year increases in interleukin-6, a proinflammatory cytokine predictive of future CAD. Furthermore, somatic symptoms may be an indicator of poor physical health or other medical conditions that may promote the development and progression of CAD. For example, sleep apnea and menopausal hormonal changes may lead to an elevation in somatic symptoms (e.g., fatigue) and contribute to the development of CAD.^[19,20]^ Our own results indicating consistently higher somatic depression symptom levels among WISE women with CAD risk factors are consistent with this previous research. Finally, it may be that there is a bidirectional relationship between somatic depression symptoms and obstructive CAD, whereby obstructive CAD can impact psychological health and well-being, while in the reverse direction, psychological factors (e.g., depression) may simultaneously influence the course of obstructive CAD through a combination of biobehavioral processes. Future studies could be designed to evaluate these possibilities.

Notably, we found that four of the seven total somatic symptoms (difficulties related to work, sleep, fatigue, and sex) were most endorsed for each group (i.e. obstructive CAD and nonobstructive CAD) and the total sample. Although we recommend assessing for somatic depression symptoms using all seven items of this subscale as the most widely validated measurement approach, providers or future researchers may consider a special focus on these four symptoms when assessing for obstructive CAD in women.

Finally, somatic depression symptoms were significantly higher among non-Caucasian participants in our sample. Although not an *a priori* prediction in our analyses, this finding is consistent with previous research observing that women from ethnic and racial minority groups are more likely to report somatic depression symptoms than Caucasian women.^[21]^ Mortality rates from CAD are also significantly higher among women from minority backgrounds,^[22]^ suggesting a need for future research to focus on improving prevention, assessment, and treatment techniques for these women.

Clinically, the results of our study suggest that a specific focus on somatic depressive symptoms could offer useful, time-efficient information about CAD risk to providers working in cardiology settings. Cardiologists, for example, may enhance their assessment of obstructive CAD by utilizing a brief measure of somatic depression symptoms that also predict CAD events. Identifying ways to improve algorithms is particularly important for women, given that traditional CAD assessments are less accurate for this group when compared to their male counterparts.^[8]^

### Study strengths and limitations

A strength of our study is that women in the WISE cohort were selected based on clinical criteria indicating a likelihood of underlying CAD. As a result, the characteristics of the cohort are very similar to women undergoing cardiology examinations in standard clinical settings. These similarities underlie the primary purpose of WISE, which was to better understand ischemic heart disease in women presenting with cardiac symptoms, including with abnormal diagnostic tests for myocardial ischemia in the absence of coronary atherosclerosis.^[8]^ Our study also contributes to the literature on depression and CAD in women, a group that has traditionally received less focus in CAD research.

Although the present study improves our understanding of the relationship between depression and CAD risk in women, there are important limitations to acknowledge. The WISE results reported in this paper were cross-sectional, increasing the possibility that nonrandomized factors related to depression, cardiac symptoms, and CAD risk could account, at least in part, for the results observed.

Recruitment methods followed for WISE were intended to reflect the usual clinical circumstances of symptomatic women undergoing assessment for the presence of CAD; however, this study characteristic limits our ability to generalize findings to women with known CAD and asymptomatic samples. Furthermore, our analyses did not include information regarding family history of coronary disease. We recognize that family history serves as an important variable, and our findings may be limited by the exclusion of this risk factor in our analyses.

Although we were able to see differences between Caucasian and non-Caucasian participants on somatic depressive symptoms, the WISE sample did not recruit a sample size of non-Caucasian participants to sufficiently power separate analyses of somatic symptoms and obstructive CAD in the latter group. This is a potentially important question for future research. In addition, we did not collect information regarding the severity of past depression, benefits or types of treatment, or the duration of the symptoms, all of which could be useful in attempts to link historical mental health data to current or prospective cardiovascular variables. Finally, the definition of hypertension changed with the 2017 American College of Cardiology/AHA Guidelines, but hypertension frequencies reported here were based on history and examination data using prior definitions, thus underestimating the prevalence by newer definitions.

## Conclusion

In a sample of women with suspected myocardial ischemia, somatic but not cognitive depressive symptoms were associated with an increased risk of obstructive CAD defined by coronary angiography. Although recommendations for general depression screening among patients with CAD already exist, our results suggest that a separate focus on somatic depressive symptoms could offer more practical and useful information relevant to CAD risk.

## Figures and Tables

**Table 1:** Somatic and cognitive items from the Beck Depression Inventory

Somatic symptoms	Cognitive symptoms
Work ability	Sadness
Sleep	Discouragement
Tiredness	Feeling like a failure
Appetite	Satisfaction
Weight loss	Guilt
Worry	Feeling punished
Libido	Disappointed in self
	Self-blame
	Suicide
	Crying
	Irritability
	Loss of interest
	Indecisiveness
	Unattractiveness

**Table 2:** Demographics, cardiac risk factors, and Beck Depression Inventory scores

	Total sample (*n*=641)	Nonobstructive CAD (*n*=427)	Obstructive CAD (*n*=214)	*T* or *χ*^2^	*P*
Age, mean (SD)	57.97 (11.38)	56.29 (10.70)	61.32 (11.96)	5.21	<0.001
Race (Caucasian) (%)	83.6	84.1	82.7	0.19	0.66
Education (>high school) (%)	41.5	44.5	35.5	4.74	0.03
Dyslipidemia (%)	52.7	43.1	71.6	43.62	<0.001
Hypertension (%)	57.2	53.9	64.0	5.90	0.02
Diabetes (%)	22.6	15.7	36.3	34.35	<0.001
Smoker status (current smoker) (%)	19.2	18.3	21.0	0.68	0.41
BMI (≥30) (%)	37.6	39.5	33.8	1.98	0.16
BDI cognitive subscale, mean (SD)	5.09 (5.92)	5.04 (5.83)	5.18 (6.10)	0.28	0.78
BDI somatic subscale, mean (SD)	5.47 (3.37)	5.23 (3.22)	5.95 (3.61)	2.48	0.01
BDI total score, mean (SD)	10.56 (8.36)	10.23 (8.12)	11.14 (8.82)	1.23	0.17
Elevated depression (BDI ≥10) (%)	44.1	43.1	46.3	0.58	0.45

BDI=Beck Depression Inventory, SD=Standard deviation, BMI=Body mass index, CAD=Coronary artery disease

**Table 3:** Women’s Ischemia Syndrome Evaluation logistic regression models adjusted for demographics

	OR*	95% CI	*P*	ROC (95% CI)
Regression 1: Somatic subscale score	1.06	1.01–1.12	0.02	0.56 (0.51–0.61)
Regression 2: Somatic subscale (low vs. high)^†^	1.43	1.02–2.02	0.04	0.56 (0.51–0.61)
Regression 3: Dyslipidemia	3.03	2.08–4.40	<0.001	0.64 (0.59–0.69)
Regression 4: Hypertension	1.34	0.94–1.91	0.11	0.55 (0.50–0.60)
Regression 5: Diabetes	3.00	2.00–4.49	<0.001	0.61 (0.56–0.66)
Regression 6: Smoking status	1.70	1.09–2.65	0.02	0.52 (0.47–0.57)
Regression 7: BMI	0.83	0.58–1.18	0.30	0.49 (0.44–0.54)

* Logistic regression models were adjusted for age, education, and ethnic status,

† A median split was used to place women into groups based on their BDI somatic subscale score. Below the median=Low group, Above the median=High group. OR=Odds ratio, CI=Confidence interval, ROC=Receiver operating characteristic, BDI=Beck Depression Inventory, BMI=Body mass index
